# Encapsulated Mn-Saturated Lactoferrin as a Safe Source of Manganese Ions for Restoring Probiotic *Lactobacillus plantarum*

**DOI:** 10.3390/molecules29122735

**Published:** 2024-06-08

**Authors:** Przemysław Gajda-Morszewski, Anna Poznańska, Eryk Federyga, Anna Ściuk, Małgorzata Brindell

**Affiliations:** 1 Department of Inorganic Chemistry, Faculty of Chemistry, Jagiellonian University in Krakow, Gronostajowa 2, 30-387 Kraków, Poland; pgmorszewski@uj.edu.pl (P.G.-M.); anna4.poznanska@student.uj.edu.pl (A.P.); eryk.federyga@student.uj.edu.pl (E.F.); 2 Doctoral School of Exact and Natural Sciences, Jagiellonian University, Prof. St. Łojasiewicza St 11, 30-348 Kraków, Poland; anna.sciuk@doctoral.uj.edu.pl; 3 Department of Crystal Chemistry and Crystal Physics, Faculty of Chemistry, Jagiellonian University in Krakow, Gronostajowa 2, 30-387 Kraków, Poland

**Keywords:** lactoferrin, manganese ions, *Lactobacillus plantarum*, encapsulation, Eudragit^®^ RS, prebiotic activity

## Abstract

The growth of *Lactobacillus plantarum*, a member of the *Lactobacillus* genus, which plays a crucial role in the bacterial microbiome of the gut, is significantly influenced by manganese ions. They can be safely delivered to the intestines by exploiting the chelating abilities of lactoferrin. The aim of this work was to encapsulate lactoferrin saturated with manganese ions (MnLf) in a system based on the Eudragit^®^ RS polymer to protect protein from degradation and manganese release in the gastric environment. The entrapment efficiency was satisfactory, reaching about 95%, and most importantly, manganese ions were not released during microparticles (MPs) formation. The release profile of the protein from the freshly prepared MPs was sustained, with less than 15% of the protein released within the first hour. To achieve similar protein release efficiency, freeze-drying was carried out in the presence of 10% (*w*/*v*) mannitol as a cryoprotectant for MPs frozen at −20 °C. MPs with encapsulated MnLf exhibited prebiotic activity towards *Lactobacillus plantarum*. More importantly, the presence of equivalent levels of manganese ions in free form in the medium, as well as chelating by lactoferrin encapsulated in MPs, had a similar impact on stimulating bacterial growth. This indicates that the bioavailability of manganese ions in our prepared system is very good.

## 1. Introduction

The lower gastrointestinal tract is colonized by a high diversity of microorganisms, with bacteria dominating. This specific ecosystem comprises around 300–500 distinct species of bacteria [1]. Among these microbial inhabitants, certain species, such as members of the *Lactobacillus* genus, are known for their beneficial effects on digestion, immune function, and overall gut homeostasis [2,3,4]. They help in protecting the host from pathogenic bacteria. The health of the host depends on the well-being of the gut microbiota. The imbalance between symbiotic and pathogenic bacteria is called dysbiosis and can cause some symptoms or be the source/cause of certain diseases [5,6]. *Lactobacillus* can modulate the intestinal epithelial lining to balance the gut barrier integrity, mucosal barrier defense, and improve host immune responses [4].

Promoting gut health depends on providing nutrients and bioactive compounds that support the growth and activity of beneficial gut bacteria. The composition of this specific microbiome is highly dependent on diet [7]. Among the essential nutrients, metal ions such as iron, zinc, and manganese play a vital role in the gut microbiota. In the case of *Lactobacillus* bacteria, manganese is essential for growth, while these bacteria can thrive in a medium with low levels of other metals, such as iron [8,9]. However, Mn, as a heavy metal, can be toxic, leading to various adverse effects on human health when exposure levels are exceeded [10]. Therefore, manganese supplementation, although beneficial in promoting the growth of *Lactobacillus* bacteria, would need to be conducted cautiously, ensuring appropriate bioavailability considering the metal’s toxicity at high concentrations. Lactoferrin (Lf), a multifunctional glycoprotein abundant in various biological fluids, has gathered considerable attention over the years for its diverse biological activities and its ability to bind and affect the bioavailability of transition metal ions (such as Fe^3+^, Zn^2+^, Cu^2+^, Mn^3+^ [11,12]).

Depending on the degree of metal saturation, lactoferrin, which offers two specific binding sites for metal ions, can exist in two forms: either saturated with metal ions, known as hololactoferrin, or depleted of metal ions with unoccupied binding sites, known as apolactoferrin (apoLf). Lactoferrin can display various properties depending on the type of metal ion bound and the degree of saturation. Apolactoferrin, for instance, can have a bacteriostatic effect on pathogenic bacteria reliant on iron by sequestering this essential element (Lf affinity to Fe^3+^ ions is very strong, with an affinity constant of 10^22^–10^23^ M^−1^ [13]). In our recent studies, we have obtained a lactoferrin complex with specifically bound Mn^3+^ ions (MnLf) [14,15] with prebiotic activity towards *Lactobacillus plantarum* and *L. rhamnosus* in vitro [14,16]. Manganese-saturated lactoferrin activity to enhance *Lactobacillus* population numbers was also confirmed using an animal model [16]. However, the population numbers of the *Lactobacillus* family did not change as remarkably as when observed in vitro. It could be caused by the fact that MnLf was administrated only for three days, and it was not protected by proteolysis in the stomach. MnLf holds promise in promoting the growth of *lactobacilli* strains through the delivery of manganese. After the release of metal ions, it may still serve as an effective iron scavenger, leveraging its higher affinity for iron to sequester this essential element and control the growth of other potentially pathogenic bacteria.

However, the therapeutic efficacy of lactoferrin is often suppressed by its susceptibility to degradation in the harsh acidic and proteolytic environment of the stomach when taken orally. Currently, researchers commonly believe that Lf cannot pass in an intact form through the proteolytic environment of the stomach. This stance is supported by both laboratory studies conducted in vitro [17,18,19,20,21] and empirical investigations carried out in vivo [22,23,24,25]. Recently, we have shown that lactoferrin saturated with iron ions undergoes complete digestion in simulated gastric fluid prepared in accordance with the recommendations of European Pharmacopoeia standards [26]. The issue of lactoferrin proteolysis in the gastric tract remains relatively underexplored. The results of experiments can be influenced by various factors such as pH levels, digestion duration, and transit time in the stomach. It should be noted that there is evidence that in some cases, a significant amount of Lf can pass through the stomach in intact form [27]. Despite these contradicting reports, even if lactoferrin successfully passes the stomach in an intact form, exposure to the acidic environment of the stomach leads to the loss of coordinated metal ions, which are crucial for its biological activity. Encapsulation of proteins is a common strategy in terms of gastrointestinal protection from degradation.

In this work, we propose the encapsulation of MnLf as a novel approach to enhance both protein and Mn bioavailability and efficacy in promoting gut health. By entrapping manganese-saturated lactoferrin within biocompatible microparticles, we aim to protect it from the proteolytic gastric environment, ensuring its safe passage to the intestines, where it can exert its beneficial effects on the gut microbiota. In this study, we utilized Eudragit^®^ RS, a member of the copolymers of the methacrylic acid family. This copolymer is pH-independent and is dedicated to microparticle formulation or coatings for slow release. Previously, this system was applied for the exceptionally effective encapsulation of bovine serum albumin (EE% = 88.4%) and horseradish peroxidase (EE% = 95.8%) [28], as well as iron-saturated lactoferrin (EE% = 90–95%) [26]. Utilizing a polymer that provides sustained release kinetics from microcapsules is advantageous, as it ensures a controlled release of contents, including manganese, which, being potentially toxic, will also be released slowly without spikes in manganese concentration levels. In vitro studies on *Lactobacillus plantarum* allowed us to determine the suitability of the proposed system containing encapsulated MnLf to deliver manganese ions, which are necessary for the growth of these bacteria.

## 2. Results and Discussion

### 2.1. Protein Encapsulation in Microparticles

**Figure 1:**
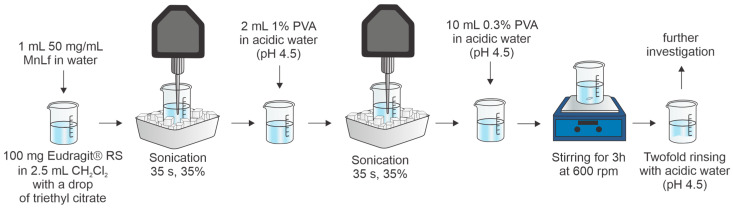
The procedure for encapsulation of manganese-saturated lactoferrin utilizing the Eudragit^®^ RS polymer.

To date, Eudragit^®^ RS has been used to encapsulate bovine serum albumin [28], horseradish peroxidase [28], and iron-saturated lactoferrin [26] using a water-in-oil-in-water double emulsion method with solvent evaporation. Recently, we have achieved an outstanding encapsulation efficiency of over 90% for iron-saturated Lf [26]. Thus, the same procedure was used to encapsulate manganese-saturated lactoferrin. The procedure is briefly outlined in the experimental section and schematically shown in Figure 1. The manganese-saturated lactoferrin (MnLf) used in this study was prepared according to the published procedure [14,15], and the resulting manganese saturation was (60.8 ± 1.4)%. The hydrodynamic radius of MnLf was (4.94 ± 0.10) nm, which closely matches the size of native lactoferrin (4.85 ± 0.10) nm. Moreover, the melting temperature, as determined by nanoDSF, indicated the higher stability of MnLf (T_m_ = 69.1 ± 0.2 °C) compared to native lactoferrin (T_m_ = 64.8 ± 0.1 °C). This observation is consistent with the high saturation level of metal ions in MnLf compared to native Lf, which is known to stabilize the structure of Lf. [29,30]. The release of the manganese ions from lactoferrin is strongly pH-dependent and accelerated by iron ions and chelating agents, as we showed in our previous work [14]. Generally, MnLf at pH 7.5 is stable, while the decrease in pH to 6.5 is enough to start releasing manganese ions.

For the analysis of the encapsulation efficiency (EE%, the ratio between the mass of the entrapped protein and the mass of total protein used for encapsulation), the supernatant above the pellet of MPs after evaporation of the organic phase was collected and analyzed for the protein content utilizing the BCA assay. The microparticles were characterized by a high encapsulation efficiency of (96.6 ± 0.8)% for MnLf, which is very close to previously reported EE% for lactoferrin saturated with iron(III) ions [26]. Moreover, the supernatant was analyzed for the presence of manganese ions by inductively coupled plasma mass spectrometry (ICP-MS). Only (4.8 ± 1.8)% of the total manganese ions were present in the supernatant, indicating that most of the manganese is located in the MPs. To obtain information on the loading capacity (LC%, the ratio of the mass of the entrapped active compound to the total mass of the MPs), the lyophilized MPs were analyzed, as they were used in this form for biological studies. Manganese determined by ICP-MS in mineralized lyophilized MPs showed that (0.26 ± 0.03) μg of Mn can be provided in 1 mg of MPs. This value corresponds to an LC% for manganese of (0.026 ± 0.003)%. Based on the amount of manganese and assuming lactoferrin saturation of ca. 61%, the theoretical protein mass was calculated to be at least (0.31 ± 0.04) mg per 1 mg of MPs, giving an LC% for the protein of (31 ± 4)%. Achieving such a high LC% for protein shows that the resulting MPs can carry very large amounts of Lf, making them a highly efficient delivery system. The data obtained suggest that the encapsulation process does not result in the loss of manganese ions and, therefore, the delivery system may find application not only in protein supply but also in manganese supplementation.

### 2.2. Release Profile—Freshly Prepared MPs vs. Lyophilized

**Figure 2:**
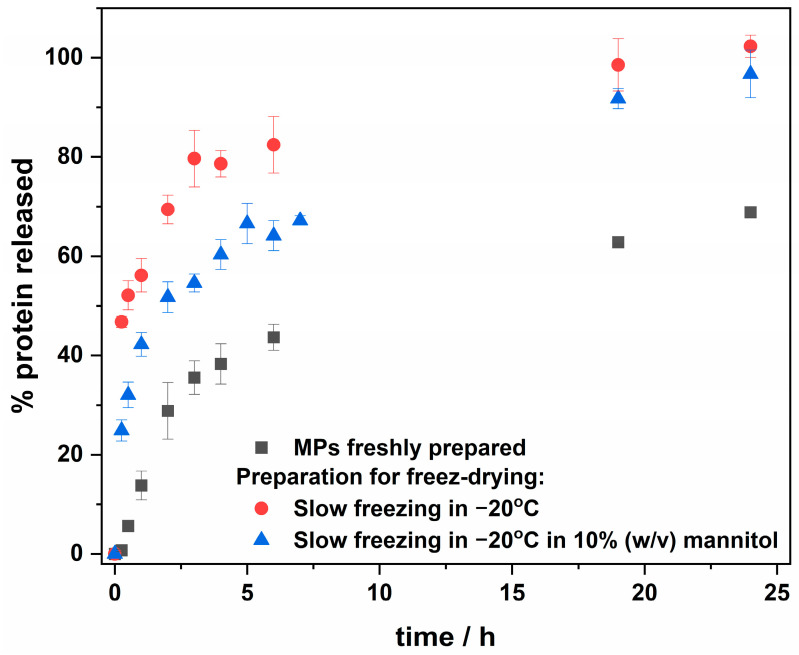
The release of lactoferrin (Lf) from freshly prepared MPs and lyophilized ones using different preparation protocols. MPs suspended in 10-times-diluted PBS pH 7.4 were placed under magnetic stirring at 300 rpm at room temperature (ca. 20 °C). The Lf concentration was determined in the supernatant from the MPs using the BCA method.

The applicability of protein encapsulation systems requires the final product to be in a solid state, easily transportable, and storable. We have recently shown that the lyophilization of MPs with encapsulated FeLf resulted in a loss of ability for controlled and slow protein release from MPs compared to the freshly prepared suspension of MPs. Therefore, a special effort was made in this study to optimize the lyophilization process. The study investigated the release profiles of MPs prepared by slow freezing at −20 °C, with or without 10% (*w*/*v*) mannitol as a cryoprotectant, followed by lyophilization. The protein release from lyophilized MPs and freshly prepared MPs is shown in Figure 2. The experiment was carried out in 10-times-diluted PBS pH 7.4 at room temperature (ca. 20 °C), and the released protein concentration was evaluated using the BCA method.

The release profile for freshly prepared MPs with encapsulated MnLf was characterized by sustained release, with less than 15% of the released protein after the first hour (Figure 2). However, MPs treated with liquid nitrogen before lyophilization released the cargo almost immediately after suspension, as observed in previous studies on encapsulated iron-saturated lactoferrin (results shown in [26]). Although a slight slowdown in the release of protein was observed with slow freezing at −20 °C, the addition of 10% (*w*/*v*) mannitol as a cryoprotectant along with slow freezing at −20 °C resulted in a sustained release of cargo from suspended MPs. Despite the increased amount of protein released within 1 h from MPs lyophilized with the addition of mannitol, compared to freshly prepared MPs, a similar amount of protein release can still be expected after a longer time. This is due to the generally greater release of protein from lyophilized MPs, reaching ca. 100% after 24 h, while for freshly prepared MPs, it reaches a maximum of 60% efficiency. Thus, freeze-drying with the addition of mannitol can therefore be considered an effective method for obtaining MPs with the desired release profile for intestinal protein delivery. Kinetic models were used to fit to the release profile data points shown in Figure 2, and the Pappas–Sahlin model (Equation (1)) [31] was found to be a good fit.

f = k_1_t^n^ + k_2_t^2n^ (1)

The fitting parameters are provided in the Supplementary Materials (Table S1, Figure S1). Analysis revealed that for freshly prepared MPs, the k_1_ constant related to Fickian diffusion is negative, suggesting that diffusion is insignificant, and that protein release is predominantly governed by the erosion and degradation of the polymer, resulting in slow and sustained release. In contrast, for MPs frozen without cryoprotectant, the k2 constant associated with polymer matrix disintegration is negative. This suggests that freezing without a cryoprotectant alters the polymer structure, causing rapid protein release, which is consistent with the observable immediate release. For MPs frozen with the addition of mannitol as a cryoprotectant, the constants are non-negative, indicating that both diffusion and polymer erosion contribute to protein release, resulting in sustained release. However, the large error in k_2_ suggests that the process may be unpredictable.

Our previous studies carried out using the same system, Eudragit^®^ RS, to encapsulate iron-saturated lactoferrin showed that for the freshly prepared MPs, the recovery of the protein kept 24 h in the simulated intestinal fluid (**IF**, 15.4 mM NaOH, 50 mM KH_2_PO_4_, pH~6.8) was ca. 80% [26]. In this study, when lactoferrin saturated with manganese ions was used, the recovery after 24 h was (96 ± 5)%. The incubation of MPs for 1 h in simulated gastric fluid (**GF**, 34.2 mM NaCl, 80 mM HCl, pH) followed by 23 h in **IF** resulted in ca. 60% recovery of FeLf [26] and (75 ± 5)% of MnLf. The observed 15% difference between both proteins, regardless of the applied conditions, may be due to using different methods for evaluating protein content. FPLC was used in the case of FeLf, while a BCA assay was used for MnLf. Previously, we also reported that for the encapsulated FeLf preincubation of protein-loaded MPs in **GF** enriched with pepsin (1 mg/mL pepsin) for 1 h, followed by incubation for 23 h in **IF,** this led to ca. 40% protein recovery, while at these conditions, FeLf alone was completely digested [26]. Therefore, it was proved that MPs protect encapsulated proteins against proteolysis.

**Figure 3:**
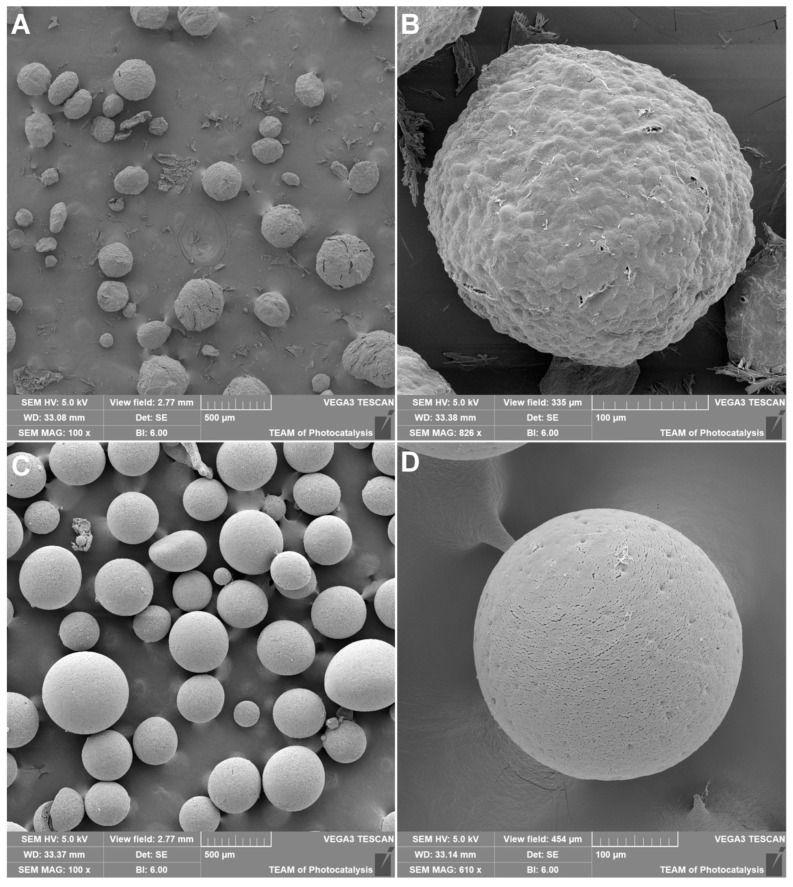
SEM images for MnLf-loaded microparticles; (**A**,**B**) MPs lyophilized in the presence of 10% of mannitol and empty; (**C**,**D**) MPs lyophilized without cryoprotectant prepared from Eudragit^®^ RS shown with different magnifications.

To further evaluate the effect of mannitol on the formulation and lyophilization of MPs, SEM imaging was conducted. MPs containing MnLf and lyophilized with 10% mannitol solution were fixed on carbon tape, coated with gold, and subjected to SEM imaging (Figure 3A,B). Additionally, empty MPs lyophilized without mannitol were imaged as per the previously described reference (Figure 3C,D) [26,28]. MPs obtained after lyophilization in the presence of mannitol have quasi-spherical shapes and visible deformation attributed to the presence of mannitol. In contrast, deformation was not apparent in empty MPs, which resembled an ideal spherical shape. The preliminary estimation of dimeters for MnLf-loaded MPs lyophilized with 10% mannitol was 274 ± 88 µm, which makes them slightly more compact than empty MPs lyophilized with the addition of cryoprotectant (304 ± 64 µm) [26]. Cryoprotectant addition positively influenced the release profile (Figure 2), as discussed above. The surface of MPs appeared smooth without cracks and pores, although larger MPs displayed cracks resulting from exposure to the electron beam and vacuum during imaging.

### 2.3. MPs Prebiotic Activity towards Lactobacillus Plantarum

A strain of *Lactobacillus plantarum* was used to test the prebiotic activity of prepared MPs with encapsulated MnLf. This strain is commonly found in the human gastrointestinal tract [32,33] and can serve as a good model for evaluating the possibility of providing manganese ions for bacterial growth. In general, bacteria are cultured in a medium (MRS broth) that already contains manganese ions, and in this work, such a complete bacterial MRS broth has been named MRS(+). To obtain information on the effect of manganese ions on the growth of *L. plantarum*, a broth depleted of manganese ions was prepared and named MRS(−). To obtain such a medium, MRS(+) was treated with Chelex^®^, using different protocols with varying incubation times and repetitions, followed by mineralization and analysis by ICP-MS to determine the manganese concentration. The best results were achieved after incubating the medium with Chelex^®^ twice (for 1 h each), which resulted in a reduction in the manganese ions concentration from (211 ± 7) μM in MRS(+) to (0.53 ± 0.03) μM in MRS(−). The application of Chelex^®^ also resulted in an approximately 30-fold reduction in the concentration of zinc ions and an approximately three-fold reduction in the concentration of magnesium ions. Both ions were added to the MRS(−) to the level before chelation to evaluate the change in the bacterial growth rate only due to the reduction in the manganese concentration.

**Figure 4:**
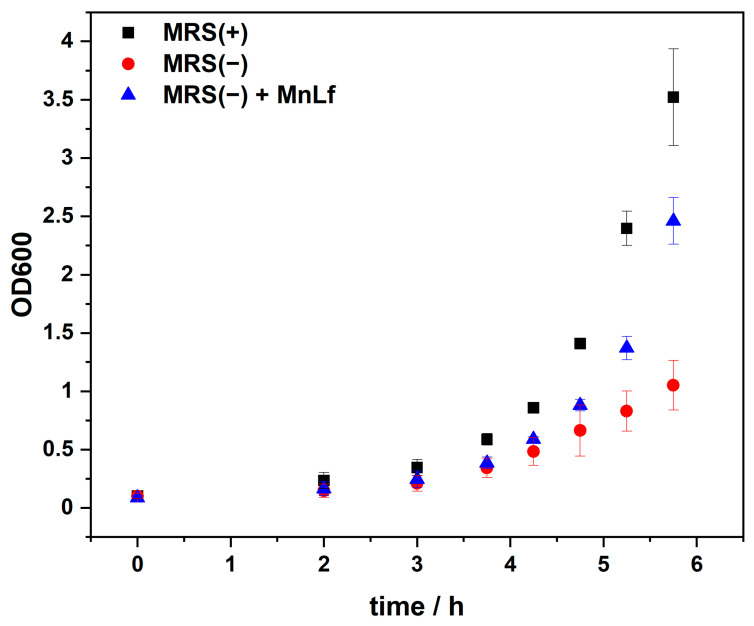
Optical density of *Lactobacillus plantarum* during incubation in a complete MRS broth, MRS(+) (■, black), Mn-depleted MRS broth, MRS(−) (●, red), MRS(−) supplemented with MnLf (▲, blue). Data are presented as mean values ± SD.

Bacterial growth was tracked by monitoring changes in the optical density at 600 nm (OD600) of *Lactobacillus* cultures. The monitoring time was set at about 6 h until the bacteria entered the logarithmic growth phase. As revealed in Figure 4, there is a distinct difference between the growth rate of *Lactobacillus* in MRS(+) and MRS(−). This confirms that bacterial growth is dependent on manganese ions and that the bacterial growth is inhibited in a medium with a negligible amount of these ions. Supplementation of the MRS(−) medium with 5 mg/mL of MnLf, equivalent to 75 µM Mn, was sufficient to accelerate bacterial growth. This concentration of MnLf was chosen based on our previous studies, showing that 5 mg/mL MnLf provides a sufficient concentration of manganese for bacterial growth, as observed in MRS(+) [14].

**Figure 5:**
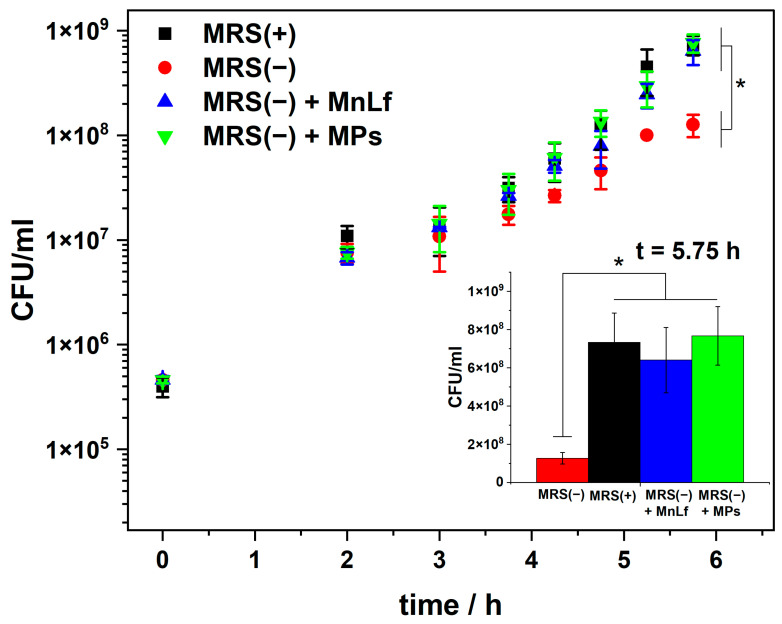
Quantitative assessment of *Lactobacillus plantarum* growth during incubation in a complete MRS broth, MRS(+) (■, black), Mn-depleted MRS broth, MRS(−) (●, red), MRS(−) supplemented with MnLf (▲ blue), and MRS(−) supplemented with MnLf encapsulated in MPs (▼, green). *Inset*: CFU counts at the time point of 5.75 h. ANOVA was used to demonstrate statistical differences between the MRS(−) and the other experimental conditions; probabilities of *p* < 0.05 (*) were considered statistically significant.

OD600 measurements were impossible to take for samples containing MPs due to the turbidity generated by decaying MPs. Therefore, CFU/mL was determined by quantitative culture on MRS agar for all conditions. The removal of manganese ions from the media affected the bacterial growth rate by slowing it down, as shown in Figure 5. This is consistent with the data obtained from the optical density measurements (Figure 4). A reduction in bacterial growth in MRS(−) medium was observed after ca. 5 h, when the bacteria entered the logarithmic growth phase. The addition of MnLf at a concentration of 5 mg/mL as a source of manganese ions to MRS(−) was sufficient to increase the bacterial growth rates similar to those observed in MRS(+), which is consistent with our previous studies [14]. To prove that MnLf encapsulated in MPs does not lose its prebiotic ability, for the test, we used lyophilized MPs prepared from a suspension slow-frozen at −20 °C without the addition of mannitol. As shown in Figure 2, such MPs release MnLf quite quickly, making it immediately available to the bacteria. Therefore, the experimental conditions used allow for a comparison of the results to be made, with those obtained with MnLf given separately. The MRS(−) was supplemented with MPs at a concentration of 40 mg/mL, resulting in a manganese concentration ca. 180–200 µM. As shown in Figure 5, MPs bearing MnLf restored the optimal bacterial growth rate with an effect similar to that of the protein given alone. This experiment confirms that the encapsulated protein can effectively deliver manganese ions to the bacteria. The obtained results show no difference in the bacterial growth rate between the complete medium and the medium after Chelex^®^ supplemented with MnLf alone or MnLf encapsulated in MPs. At the same time, the difference between MRS(−) and all other conditions is evident and can be considered statistically significant for the measurement taken after 5.75 h (Figure 5, inset).

### 2.4. Toxicity of MPs—Preliminary Studies

**Figure 6:**
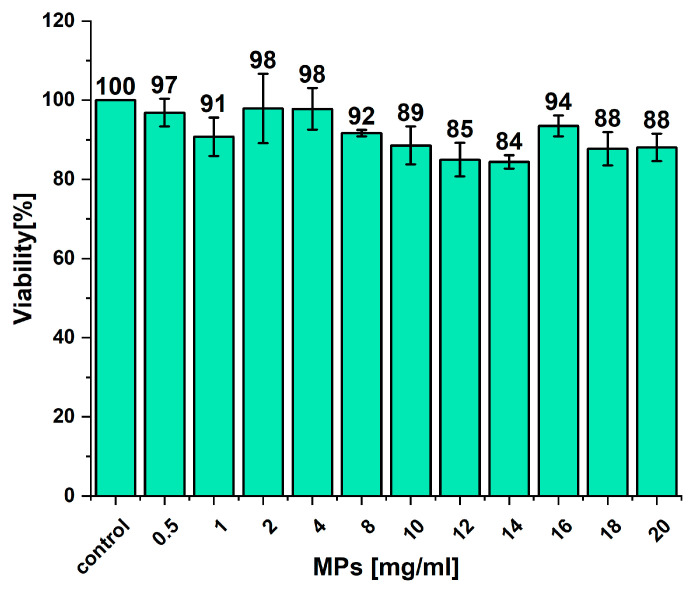
Viability of HT-29 cells after incubation with microparticles prepared from Eudragit^®^ RS for 24 h. Data are presented as mean values ± SD, and 100% viability was assigned to the untreated cells and referred to as control.

The toxicity of empty Eudragit microparticles and microparticles bearing BSA was previously assessed using the human intestinal epithelial colorectal adenocarcinoma cell line (CaCo2), and they were found to not be toxic in a wide range of concentrations up to 20 mg/mL [28]. In this study, we also performed a similar experiment using a human colon epithelial colorectal adenocarcinoma cell line (HT-29), and as shown in Figure 6, the empty MPs are nontoxic even at concentrations as high as 20 mg/mL. The obtained data confirm previous findings. Additionally, Eudragit is approved by the FDA and EMA as a polymer for tablet/capsule formulations and coating agents, indicating its safety. Concerning the cargo of MPs, manganese-modified lactoferrin, both in vitro and in vivo studies on mice, showed a lack of toxicity of this protein [16].

## 3. Materials and Methods

Eudragit^®^ RS was kindly donated by Evonik Industries AG (Essen, Germany). In this study, we used bovine lactoferrin from Mercurius Production GmbH (Frankfurt, Germany), and it was called native lactoferrin. Polyvinyl alcohol (PVA) MW 85,000–124,000 g/mol, 99+% hydrolyzed, was purchased from Sigma-Aldrich (Saint Louis, MO, USA). All other chemicals were purchased from Sigma-Aldrich or Avantor (Gliwice, Poland) and were of at least analytical grade. MiliQ class water (Merck Millipore system, Darmstadt, Germany) was used to prepare all aqueous solutions.

### 3.1. Metal Content Determination

Metal concentrations were determined by inductively coupled plasma mass spectrometry (ICP-MS). A collision-reaction cell was employed along with a kinetic energy discrimination (KED) technique, utilizing helium as an inert gas, to eliminate polyatomic interferences. All samples were mineralized in concentrated nitric acid (Sigma-Aldrich, trace metal basis, ≥99.999%) for 24 h at 60 °C and then diluted in MiliQ grade water to 1% of acid and subjected to analysis using an ICP-MS spectrometer (Perkin Elmer NexION 2000C, Waltham, MA, USA).

### 3.2. Saturation of Lactoferrin with Manganese (MnLf)

Metal-free lactoferrin (apoLf) and manganese-saturated lactoferrin (MnLf) were obtained according to a published procedure [14]. Briefly, native Lf (from Mercurius Production GmbH, Frankfurt, Germany) was dialyzed three times in 0.1 M citric buffer, pH 3.5 (each dialysis for 3 h), to remove iron, followed by dialysis in water. The resulting apoLf was dialyzed at 37 °C for 24 h in 50 mM HEPES, 100 mM NaCl, 25 mM NaHCO_3_, pH 7.4 enriched with MnCl_2_, given in a twenty-fold excess relative to lactoferrin. The manganese saturation level in Lf was determined by ICP-MS, as described in Section 3.1, and was (60.8 ± 1.4)%. Stability measurements for native lactoferrin and MnLf were performed on Prometheus Panta (NanoTemper Technologies GmbH, München, Germany). The hydrodynamic radius of the protein was measured using DLS mode, while the melting temperature was analyzed by nanoDSF mode (nano-differential scanning fluorimetry).

### 3.3. Protein Encapsulation

Eudragit^®^ RS-based microparticles (MPs) containing MnLf were obtained using the previously described protocol for the encapsulation of iron-saturated lactoferrin [26]. Briefly, MnLf (10 or 50 mg) was dissolved in pure MiliQ H_2_O and sonicated with 100 mg of Eudragit^®^ RS dissolved in 2.5 mL of CH_2_Cl_2_ with one drop of triethyl citrate (Avantor). The Q500 Sonicator^®^ (Newton, CT, USA) equipped with a 1/8″ probe was used to obtain the first emulsion (water-in-oil) by applying sonication for 35 s with an amplitude of 35%; emulsion was kept on an ice bath during sonication. The initial emulsion was sonicated with 2 mL of 1% (*w*/*v*) PVA in acidic solution (pH~4.5, acetic acid) to produce the final water-in-oil-in-water emulsion. In addition, 10 mL of 0.3% (*w*/*v*) PVA (in acidic solution) was added to ensure stability, and the emulsion was stirred at 600 rpm for 3 h to allow the dichloromethane to evaporate. The MPs were then washed three times with acidic water (pH~4.5, acetic acid) and collected by decantation (the protocol is shown schematically in Figure 1). For further experiments, freshly prepared MPs were used or lyophilized. Prior to lyophilization, MPs were frozen at −20 °C alone or with the addition of 10% (*w*/*v*) mannitol as a cryoprotectant. The alpha 1–2 LDplus freeze-dryer (Christ, Osterode am Harz, Germany) was used for lyophilization at a vacuum of 0.31 mbar for 18 h. EE% was calculated by the determination of unencapsulated protein in the supernatant above the MPs by applying the BCA assay with the Pierce BCA Protein Assay Kit (23225, Thermo Fisher Scientific, Waltham, MA, USA) according to the manufacturer’s protocol. In addition, the supernatant was mineralized, and manganese was determined by ICP-MS, as described in chapter 3.1. The manganese content was also determined in lyophilized MPs (without mannitol), which were used for in vitro studies to obtain the exact amount of manganese per 1 mg of MPs by ICP-MS. The morphology of the MPs after lyophilization in the presence of mannitol was studied by SEM. Lyophilized MPs were fixed onto carbon tape and coated with a thin layer of gold (Quorum Q150R coater) and subjected to SEM imaging using Tescan VEGA 3 microscope (Tescan Orsau Holding a.s., Brno, Czech Republic). For estimation of the size of the MPs, the ImageJ 1.53e program was applied [34,35]. The diameter was calculated based on 50 objects from the MPs sample.

### 3.4. Protein Release Profile Investigation

The protein release profile was studied in tenfold diluted PBS, pH 7.4 (Corning, NY, USA). The assay was performed for freshly obtained MPs and lyophilized MPs. MPs were prepared for the lyophilization by slow freezing at −20 °C with or without the addition of mannitol as a cryoprotectant. MPs suspended in PBS were incubated at room temperature on a magnetic stirrer (300 rpm). The release profile was studied for 24 h. At specific time points, aliquots of the suspension were taken and centrifuged (10 min 5000 rpm), and the concentration of released protein was determined using the BCA method. The percentage of released protein was calculated as the mass of released protein relative to the total mass of protein in the MPs (calculated from the mass of the MPs and the LC% determined at the characterization step).

The protein release from MPs was also studied under conditions designed to simulate the gastrointestinal tract. On the basis of the 10th edition of the European Pharmacopoeia, simulated gastric fluid (GF, 34.2 mM NaCl, 0.08 M HCl, pH~1.1) and simulated intestinal fluid (IF, 15.4 mM NaOH, 50 mM KH_2_PO_4_, pH~6.8) were prepared. MPs (freshly formulated) were washed three times with GF, resuspended in a fresh portion of GF, and incubated at room temperature under stirring at 300 rpm for 1 h. The supernatant was collected after 1 h, and the remaining MPs were washed three times with IF, resuspended in a fresh aliquot of IF, and incubated for 23 h. After 23 h, an aliquot of the supernatant was collected, and the released protein was determined by the BCA method. As a reference, to monitor total release without pH-dependent protein denaturation, MPs were resuspended in IF, and an aliquot of the supernatant was collected after 24 h. In addition, the remaining MPs were collected after each experiment and sonicated to obtain full disintegration of the MPs and complete protein release. All samples were centrifuged (14,000× *g* for 30 min), and the collected supernatants were analyzed using the BCA method.

### 3.5. Bacteria Cultivation

The prebiotic activity of the obtained MPs containing MnLf was tested on a strain of *Lactobacillus plantarum* subsp. *plantarum* (ATCC 14917, American Type Culture Collection, Manassas, VA, USA). Bacteria were passaged on MRS agar plates (VWR), and the plates were incubated at 37 °C and 5% CO_2_ atmosphere. As a competent medium for bacterial cultivation, a ready-to-dissolve mixture of MRS from Sigma-Aldrich (52.2 g per 1 L of MilliQ H_2_O) was utilized; in this paper, it is referred to as MRS(+). The medium was sterilized in an autoclave according to the manufacturer’s specifications, i.e., 15 min at 121 °C.

MRS(+) medium has a high concentration of Mn, which is essential for the optimal growth of *Lactobacillus* bacteria. To study the effect of encapsulated MnLf on bacterial growth, it was necessary to prepare a medium depleted of Mn. The medium was incubated with the ion exchange resin Chelex^®^ 100 specific to divalent ions (50 g of resin per 1 L of medium) at room temperature for 1.5 h. After this time, the medium was filtered through filter paper, and the incubation was repeated with a fresh portion of Chelex^®^ (a single incubation proved insufficient to remove the appropriate amount of Mn). The prepared medium depleted of divalent ions, including Mn, was supplemented with MgCl_2_ and ZnSO_4_ to obtain concentrations present in MRS(+) of [Mg^2+^] = 1 mM and [Zn^2+^] = 30 µM, respectively, and then sterilized in an autoclave. The concentrations of metals in the media were monitored by ICP-MS.

Bacterial suspensions for the experiments were prepared by suspending a single bacterial colony in complete MRS(+) broth (Sigma-Aldrich) one day before the experiment and cultured in 37 °C and 5% CO_2_ atmosphere overnight. Bacteria were collected the next day by centrifugation (3500× *g* 5 min 10 °C), washed three times with PBS without Mg^2+^ and Ca^2+^, resuspended in the tested broth (MRS(+), MRS(−), MRS(−) + MnLf), and diluted to OD600 = 0.1 for the experiment. OD600 measurements were performed using a Perkin Elmer Lambda 265 spectrophotometer (Waltham, MA, USA) in a cuvette with a 1.00 cm optical path length.

The effect of MPs with encapsulated MnLf on the growth of *lactobacilli* was investigated by suspending the MPs in a bacterial suspension with OD600 = 0.1 in MRS(−). The experiment was conducted at 37 °C with gentle shaking at 200 rpm. At appropriate time points, the supernatant was collected from above the MPs and a dilution series was made and seeded quantitatively on MRS agar to quantify the bacteria. As a positive control, experiments were also conducted for bacterial suspensions with an initial OD600 = 0.1 in MRS(+) and as a negative control in MRS(−). In addition, measurements were carried out for cultures in MRS(−) enriched with 5 mg/mL MnLf.

### 3.6. Toxicity Studies

HT-29 cells were cultured in McCoy’s medium supplemented with 10% fetal bovine serum (FBS) until reaching confluence and maintained at 37 °C in a designated cell culture incubator with 5% CO_2_. Subsequently, the cells were harvested by trypsinization, counted, and seeded into 24-well plates at a density of 35,000 cells per well in McCoy’s medium (with FBS), followed by overnight incubation. The following day, the cells were washed with phosphate-buffered saline (PBS) containing Mg^2+^ and Ca^2+^, and suspensions of the investigated microparticles (MPs) were added to the cells in McCoy’s medium without FBS. The cells were then incubated for an additional 24 h, after which viability was assessed using the resazurin assay. Experiments were performed in duplicate.

## 4. Conclusions

The encapsulation of MnLf in MPs formed using Eudragit^®^ RS was highly efficient and did not result in the release of manganese ions during the process. The optimization of the freeze-drying process of the MPs enabled the sustained release of protein saturated with manganese ions. A total of 1 mg of lyophilized MPs can deliver ca. 0.26 μg of manganese. The beneficial aspect of using MnLf encapsulated in MPs is not only the protection of the protein from gastric digestion but also the controlled release of potentially harmful manganese ions. This controlled release prevents spikes in manganese concentrations that could lead to adverse effects. We have demonstrated that the efficient delivery of manganese ions promotes the growth of *Lactobacillus plantarum*, a manganese-dependent probiotic lactic acid bacterium. The studied system could be recommended for further testing as an effective prebiotic to enhance the growth of *Lactobacillus*, which helps to restore the gut flora after antibiotics or chemotherapy.

## Data Availability

The data presented in this study are available in the main text and Supplementary Materials.

## Supplementary Materials

The following supporting information can be downloaded at: https://www.mdpi.com/article/10.3390/molecules29122735/s1. Figure S1. Peppas-Sahlin kinetic model fit to the release of lactoferrin; Table S1. The summary of the fitting parameters for Pappas-Sahlin model.
