# Supplementary material for: Encapsulated Mn-Saturated Lactoferrin as a Safe Source of Manganese Ions for Restoring Probiotic Lactobacillus plantarum
Source: Molecules. 2024 Jun 8;29(12):2735. doi: 10.3390/molecules29122735 (PMC11205955; doi:10.3390/molecules29122735)
Supplement: Supplementary file 1 [file molecules-29-02735-s001.zip › molecules-2959262-supplementary.pdf]

Encapsulated Mn-saturated lactoferrin as a safe source of manganese ions for restoring probiotic *Lactobacillus plantarum*

Przemysław Gajda-Morszewski [1,2], Anna Poznańska [1], Eryk Federyga [1], Anna Ściuk [2,3] and Małgorzata Brindell [1,*]

[1] Department of Inorganic Chemistry, Faculty of Chemistry, Jagiellonian University in Krakow, Gronostajowa 2, 30-387 Kraków, Poland

[2] Doctoral School of Exact and Natural Sciences, Jagiellonian University, Prof. St. Łojasiewicza St 11, 30-348 Kraków, Poland

[3] Department of Crystal Chemistry and Crystal Physics, Faculty of Chemistry, Jagiellonian University in Krakow, Gronostajowa 2, 30-387 Kraków, Poland

* Correspondence: malgorzata.brindell@uj.edu.pl

Supplementary Material Section

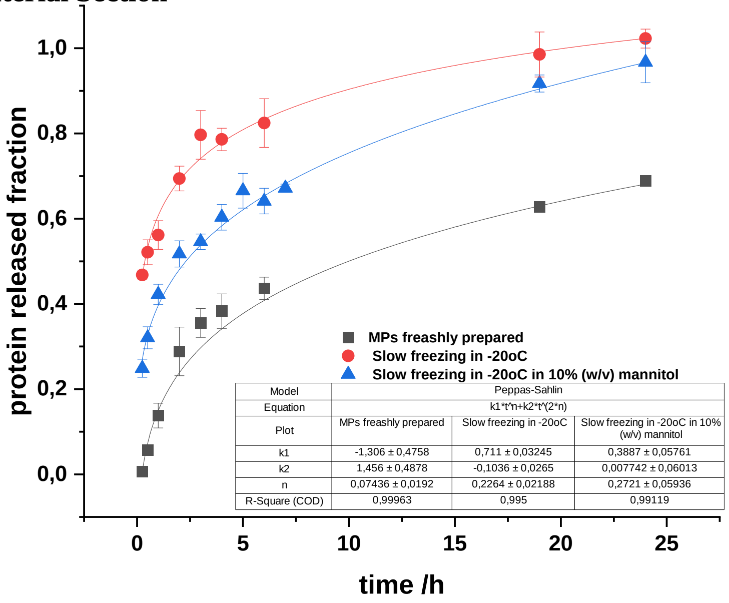

Figure S1. Peppas-Sahlin kinetic model ($f = k_1t^n + k_2t^{2n}$) fit to the release of lactoferrin (Lf) from freshly prepared MPs and lyophilized ones using different preparation protocols. MPs suspended in 10 times diluted PBS pH 7.4 were placed under magnetic stirring at 300 rpm at room temperature (ca. 20 °C). The Lf concentration was determined in the supernatant from the MPs using the BCA method.

Table S1. The summary of the fitting parameters for Pappas-Sahlin model.

Parameter	MPs freshly prepared	MPs lyophilized after slow freezing -20 °C	MPs lyophilized after freezing with mannitol 10% (w/v)
k_1	-1.3 ± 0.5	0.71 ± 0.03	0.39 ± 0.06
k_2	1.5 ± 0.5	-0.10 ± 0.03	0,008 ± 0,060
n	0.07 ± 0.02	0.23 ± 0.02	0.27 ± 0.06
